# Association among social support, self-identity, depression, and anxiety in college student men who have sex with men in Guiyang, China

**DOI:** 10.3389/fpsyg.2026.1911298

**Published:** 2026-10-08

**Authors:** Yong Lu, Kai Qin, Jiao Chen, Lu Ma, Bingyi Wang, Bo Yan, Di Liu

**Affiliations:** 1 Key Laboratory of Environmental Pollution Monitoring and Disease Control, Ministry of Education, School of Public Health, Guizhou Medical University, Guiyang, China; 2 Guiyang Center for Disease Control and Prevention (Guiyang Health Supervision Institute), Guiyang, China; 3 Institute of HIV/AIDS Control and Prevention, Guangdong Provincial Centre for Disease Control and Prevention, Guangzhou, China; 4 Department of HIV/AIDS Control and Prevention, Guangdong Provincial Academy of Preventive Medicine, Guangzhou, China; 5 Guangzhou Medical University Library, Guangzhou, China; 6 School of Public Health, Guangzhou Medical University, Guangzhou, China

**Keywords:** anxiety, China, college student, depression, men who have sex with men, self-identity, social support

## Abstract

**Background:**

This study aimed to investigate depression and anxiety among college student men who have sex with men (MSM) and to explore their associations with social support and self-identity, with a particular focus on the mediating role of self-identity.

**Methods:**

A cross-sectional study was conducted among college student MSM in Guiyang, China, from September 2022 to October 2023. We used a self-administered questionnaire to collect information about demographic characteristics, social support, self-identity, depression, and anxiety. Structural equation modeling (SEM) was applied to investigate the associations among social support, self-identity, depression, and anxiety. The bootstrapping method was performed to examine the mediating role of self-identity.

**Results:**

A total of 547 valid questionnaires were collected. In this study, 38.39% of the participants were depressed and 28.34% were anxious. The results revealed that social support (β = −0.110, *P* = 0.030) and self-identity (β = −0.253, *P* = 0.001) were negatively related with depression, whereas anxiety (β = 0.161, *P* = 0.008) was positively associated with depression. Lower social support and self-identity were linked with higher anxiety, with social support showing a β = −0.107 (*P* = 0.017) and self-identity showing a β = −0.252 (*P* < 0.001). Also, social support had a positive relationship with self-identity (β = 0.112, *P* = 0.004). Furthermore, the results showed that self-identity served as a mediator in the relationship between social support and depression (β = −0.028, *P* = 0.010). Additionally, self-identity also played a mediating role in the relationship between social support and anxiety (β = −0.028, *P* = 0.011).

**Conclusions:**

College student MSM in Guiyang exhibited mild depressive symptoms and mild-to-moderate anxiety. Compared to the general MSM population in China, the prevalence rates of anxiety and depression among college student MSM were relatively lower. These findings underscore the continued need for interventions aimed at reducing depression and anxiety among college student MSM. Enhancing both social support and self-identity may represent effective strategies for mitigating anxiety and depression in this population.

## Introduction

In recent years, studies focusing on mental health problems among men who have sex with men (MSM) have increased rapidly. MSM include homosexual, bisexual, transsexual, and heterosexual men who have ever had sex with men (Zhang et al., 2023). MSM are more likely to experience mental health problems, especially depression and anxiety, compared to the heterosexual population (Liu and Jiang, 2022). A previous meta-analysis reported that the global prevalence of depression among MSM was 35.0% (Nouri et al., 2022), and an internet-based survey reported an anxiety prevalence of 23.4% among MSM (Parker et al., 2015). Mental health problems are also severe among Chinese MSM. A meta-analysis revealed that 43.2 and 32.2% of Chinese MSM reported depressive and anxiety symptoms, respectively (Wei et al., 2020). The Minority Stress Theory posits that MSM are at a high risk of depression and anxiety due to facing various external stressors (e.g., social prejudice and discrimination) and internal stressors (e.g., self-identity and internalized homophobia) (Meyer, 2003). Additionally, the traditional Chinese culture is different from that of Western countries. Neither law nor social values in China support same-sex marriage or partnership. Such sociocultural factors create a heteronormative, stigmatizing environment that exacerbates depression and anxiety among Chinese MSM (Zhang and Chu, 2005; Liu et al., 2018).

Apart from the above-mentioned stressors, studies concentrating on college student MSM have reported various other stressors they encountered. Compared to general college students, college student MSM are more likely to encounter academic pressure and financial pressure, which are related to greater severity of depression and anxiety (Gorman and Charvis, 2024; Parchem et al., 2024). A survey conducted among sexual minority college students in the United States indicated that bullying victimization might occur during the college period, and it was positively associated with depression symptoms (Moran et al., 2018). Furthermore, in recent years, college student MSM in China have emerged as one of the key high-risk populations for HIV infection (Li et al., 2013). The perceived risk of HIV infection has been confirmed to have a strong association with depression and anxiety (Hsieh, 2013). Research conducted among college student MSM in Chengdu, China revealed that the prevalences of anxiety (50.6%) and depression (81.3%) were substantially higher than those observed in the general MSM population (Hongyu et al., 2022). Therefore, it is of great value to pay attention to depression and anxiety among college student MSM in China.

Social support has been widely demonstrated to be a positive factor associated with depression and anxiety among sexual minorities (Shao et al., 2018; Pan et al., 2022; Li et al., 2023). Social support is defined as “one's perception of different kinds of support (including physical, financial, and psychological support) that he/she can receive from family members, peers, and other members in the network in times of need” (Cohen and Wills, 1985). A literature review has indicated that increased social support is associated with lower levels of depression and anxiety among sexual minority adolescents (Mcdonald, 2018). Similar results were found among college student MSM in China (Xie et al., 2024).

Moreover, another important factor related to depression and anxiety is self-identity. Self-identity refers to the individual's perception and internalization of their role (De Bruijn et al., 2012). Previous study suggested that self-identity incongruence was associated with increasing levels of depression and anxiety. A cross-sectional study conducted in Taiwan showed that MSM with self-identity disturbance were at higher risk of depressive and anxiety symptoms (Chen et al., 2021). Meanwhile, self-identity was found to be a predictor that could negatively predict levels of depression and anxiety among college students (Li et al., 2022). However, to the best of our knowledge, no existing research has explored the association between self-identity and both depression and anxiety among college student MSM.

Several previous studies of sexual minorities have found that social support was a facilitative factor for self-identity development (Shilo and Savaya, 2011; Brandon-Friedman and Kim, 2016; Xu et al., 2023). Individuals who received more social support were more likely to have a positive self-identity, which was positively related to mental wellbeing (Vransisca, 2022). Several empirical studies have also confirmed this theory. A study conducted among college students indicated that social support could influence depression and anxiety through self-identity during the COVID-19 pandemic (Li et al., 2022). There was similar evidence among sexual minorities (Lin et al., 2022). However, among college student MSM, it remains unclear whether self-identity mediates the associations of social support with both depression and anxiety.

There has been no research on depression and anxiety among college student MSM in Guiyang. Although self-identity has been identified as an important predictive factor of depression and anxiety among MSM, college student MSM are a distinct subgroup that remains understudied. The associations of self-identity with depression and anxiety in this population remains unexamined. Furthermore, while previous studies have revealed associations of social support with both depression and anxiety among college student MSM, whether self-identity mediates these associations remains underexplored. Therefore, the purposes of this study are to (1) explore depression and anxiety among college student MSM in Guiyang; (2) to examine the associations among social support, self-identity, depression, and anxiety among college student MSM; (3) to examine whether self-identity mediates the associations of social support with both depression and anxiety.

### Research hypotheses and theoretical basis

Integrating the theoretical perspectives and empirical evidence, we propose the following hypotheses (Figure 1):

**Figure 1:**
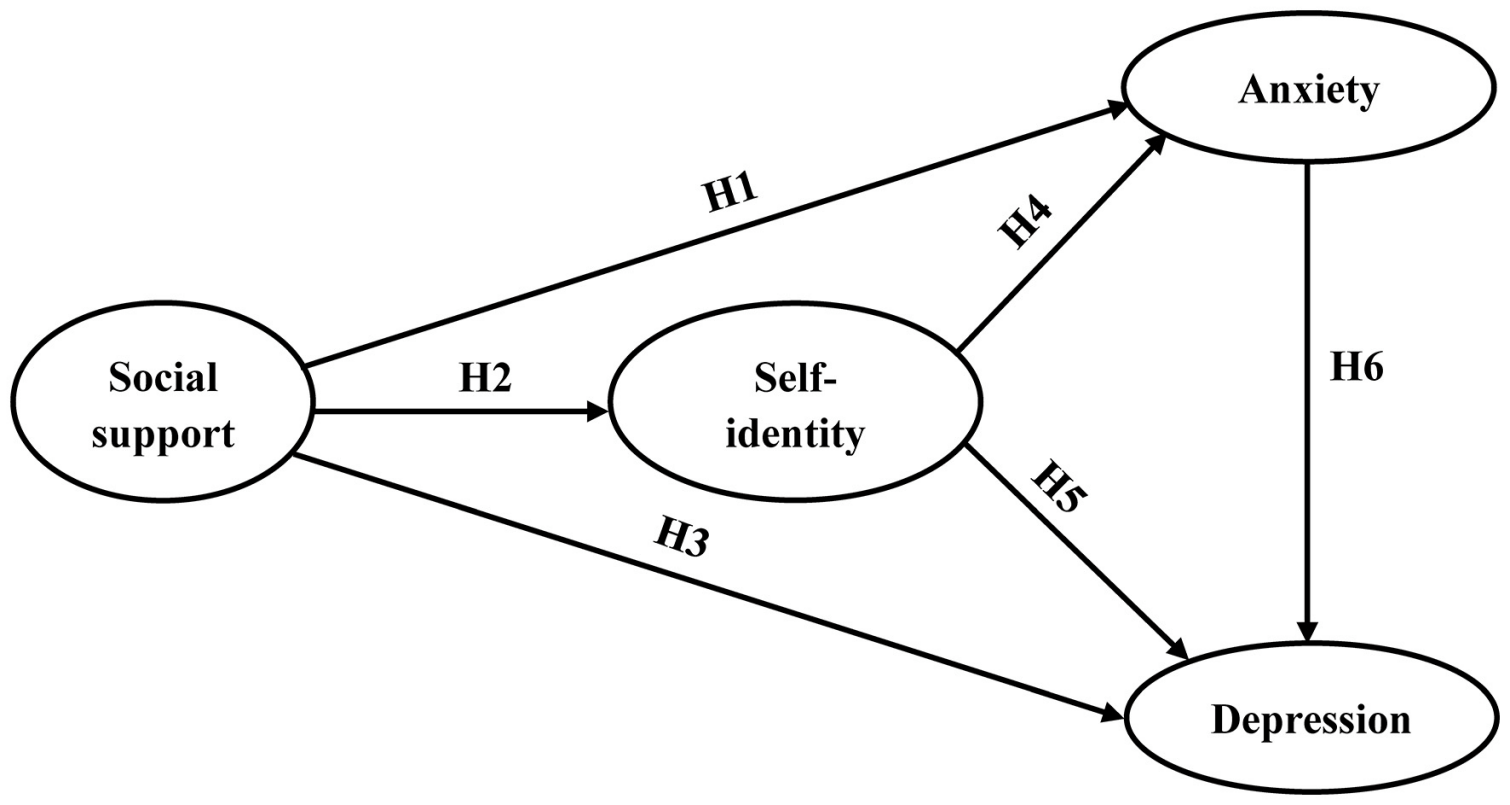
Hypothesized model of associations among social support, self-identity, depression, and anxiety in college student MSM.

H1: Social support is negatively associated with anxiety.

H2: Social support is positively associated with self-identity.

H3: Social support is negatively associated with depression.

The three hypotheses are based on the minority stress model. This model posits that college student MSM face unique, chronic stressors (such as stigma, discrimination, and identity concealment) that increase their risk of depression and anxiety (Tan et al., 2022; Chen et al., 2026). Within this framework, social support is viewed as a crucial buffer, providing emotional, practical, and informational resources that help MSM individuals cope with these stressors. Specifically, robust social support networks may reduce anxiety by providing reassurance and help in dealing with discrimination, while also building positive self-identity by affirming one's sexual orientation and creating a sense of belonging and acceptance (Zhou et al., 2025). By reducing the psychological burden of chronic stress and enhancing self-worth, social support is therefore expected to be associated with lower levels of depression (Xie et al., 2024; Chen et al., 2026). These hypothesized relationships are well-supported by empirical evidence among MSM populations (Tan et al., 2022; Xie et al., 2024; Guo et al., 2025; Zhou et al., 2025; Chen et al., 2026).

H4: Self-identity is negatively associated with anxiety.

H5: Self-identity is negatively associated with depression.

These hypotheses propose that self-identity is negatively related to both anxiety and depression among MSM populations. The theoretical basis comes from the idea that having a strong and positive self-identity provides individuals with important psychological resources to cope with the unique challenges associated with being a sexual minority. For MSM, who often face prejudice, discrimination, and internalized negative social attitudes, a solid sense of self-identity (including self-esteem, being true to oneself, and positive group belonging) can theoretically protect against the mental health effects of these experiences, thereby reducing the risk of internalizing problems (Meyer, 2003). Empirical evidence also supports this view, as identity struggles and stigma among MSM are linked to higher depression and anxiety (Pascoe and Smart Richman, 2009), and self-identity confusion has also been reported to be associated with anxiety and social difficulties (Hatzenbuehler, 2009). Therefore, it is reasonable to propose that self-identity is negatively associated with anxiety and depression.

H6: Anxiety is positively associated with depression.

This hypothesis posits a direct and positive correlation between anxiety and depression among MSM. This is based on the idea that both conditions share common risk factors, such as minority stress from stigma and discrimination, which can trigger both anxious and depressive feelings. Prior studies have consistently found that anxiety and depression often co-occur in MSM populations, with higher anxiety levels tied to greater depressive symptoms (Wu et al., 2023; Zheng et al., 2025). Therefore, it is reasonable to hypothesize that anxiety is positively associated with depression.

## Methods

### Study population and procedure

From September 2022 to October 2023, we conducted a cross-sectional study in Guiyang, China. Considering the dispersed and hidden nature of college student MSM, we recruited participants using three methods: online, venue-based, and snowball recruitment. Inclusion criteria of participants included: (1) male, (2) college students in Guiyang City, (3) had oral and/or anal sex with other males in the past 12 months, and (4) signed informed consent. Exclusion criteria were MSM with: (1) cognitive impairment, (2) communication disorders, or (3) self-identification as heterosexual. All eligible participants were asked to complete a self-structured questionnaire anonymously in a separate room. Then, trained investigators checked the completeness and logical consistency of the completed questionnaires to ensure data quality. Ethics approval was obtained from the Ethics Committee of Guizhou Medical University [Approval No. 2023(43)].

### Measurements

#### Socio-demographic information

We collected the following socio-demographic information from all included participants: age, hometown (Guiyang or other cities), ethnicity (Han or others), stage of study (junior college/undergraduate or master/doctor), major (medical or non-medical), living arrangement (on-campus or off-campus), living expenses per month (<¥2,000 or ≥¥2,000), and student loans (yes or no).

#### Depression

The Center for Epidemiological Studies Depression Scale (CES-D) was adopted to evaluate depressive symptoms (Radloff, 1977). This scale consists of 20 items, and each item asks participants to rate how often they had experienced different depressive symptoms in the past 7 days using a four-point Likert scale with “0” representing “rarely or none of the time” and “3” representing “most of the time or persistent”. Four items related to positive perceptions are reverse scored. The total score of depression is the sum of the scores for each item. Individuals with a score ≥10 are classified as depressed (Moon et al., 2017). The higher the total score, the higher the level of depression. CES-D has been extensively applied and validated in a lot of previous Chinese studies (Niu et al., 2021; Zhu et al., 2021). In our study, the Cronbach's alpha value for CES-D was 0.849, indicating good internal reliability.

#### Anxiety

In this study, anxiety symptoms were assessed with the Self-rating Anxiety Scale (SAS) (Dunstan and Scott, 2020). This scale contains 20 items that cover various anxiety symptoms. Participants are asked about the frequency of different anxiety symptoms in the past 7 days. All items are rated on a four-point Likert scale ranging from 1 (None or very little time) to 4 (Most or all the time). Five items are reverse-scored. The standard score is calculated by summing the scores of each item and then multiplying by 1.25. Individuals with a score ≥45 are identified as anxious (Zung, 1986). A higher standard score indicates more severe anxiety symptoms. SAS has been widely used in China and proved to be a reliable and valid scale (Cao et al., 2023). The Cronbach's alpha value obtained in our study was 0.805, suggesting good internal reliability.

#### Social support

We measured the level of social support with the Social Support Rating Scale (SSRS) (Shuiyuan, 1994). The scale consists of 10 items encompassing the following three dimensions: objective social support, subjective social support, and utilization of social support. Objective social support represents the support that is objective, visible, and realistic, including direct material help and the presence and participation of the social network. Subjective social support refers to subjective feelings and emotional support, including emotional experience and the perception of being supported, respected, and understood. The utilization of social support refers to the extent to which participants seek, use, or participate in social support. Scores from each item were accumulated to give a total score. The higher the total score, the better the social support. Previous studies conducted in China have shown the high reliability and validity of the SSRS (Zhao et al., 2021; Liu et al., 2022). In the current study, the Cronbach's alpha values for the three dimensions ranged from 0.663 to 0.745, indicating moderate internal reliability.

#### Self-identity

The Self-identity as Homosexual Scale (Guo-Zheng et al., 2012), which was jointly developed by the Shanghai Center for Disease Control and Prevention and the Dalla Lana School of Public Health, University of Toronto, was used to determine the level of self-identity among MSM. This scale comprises nine items related to self-identity as a male homosexual (e.g., sometimes I wish I were not gay). All items are scored on a 5-point Likert scale, ranging from 1 (strongly agree) to 5 (strongly disagree). Item 9 (i.e., I am happy that I am gay) is reverse scored. The total score is obtained by summing the scores for all items. The higher scores indicate a more positive self-identity as a male homosexual. This scale has been shown to have satisfactory reliability (Guo-Zheng et al., 2012). In our study, a Cronbach's alpha of 0.860 also indicated good internal reliability.

### Statistical analyses

We calculated descriptive statistics for socio-demographic information, depression, anxiety, social support, and self-identity. Continuous variables were described using mean and standard deviation (SD), and categorical variables were presented in frequencies and percentages. Given that the data for depression, anxiety, social support, and self-identity did not satisfy the assumption of bivariate normality, Spearman's correlation analysis was used to examine the correlations among these variables. Then, a structural equation model (SEM) was applied to explore the associations among social support, self-identity, depression, and anxiety, and to test the mediating role of self-identity while controlling for the socio-demographic factors listed in Table 1. To test the significance of the mediation, we employed a bootstrapping procedure with 2,000 iterations, estimating 95% confidence intervals (CIs). We used the following indicators to measure the goodness-of-fit of the SEM: variance divided by degrees of freedom (χ^2^/df), root mean square error of approximation (RMSEA), incremental fit index (IFI), normed fit index (NFI), Tucker-Lewis incremental fit (TLI), adjusted goodness of fit index (AGFI), comparative fit index (CFI), and goodness of fit index (GFI). The following reference values were adopted as indices for adequate model: χ^2^/df <5, RMSEA less than 0.08, AGFI greater than 0.80, and IFI, NFI, TLI, CFI, and GFI all >0.90 (Kim et al., 2019). Descriptive analysis and correlation analysis were conducted using SPSS 20.0, and the SEM analysis using the maximum-likelihood estimation was performed by SPSS AMOS 26.0. Two-tailed *P* value ≤ 0.05 was considered statistically significant.

**Table 1:** Socio-demographic information of college student MSM in Guiyang.

Variable	*N*	Percentage (%)
Age		
<18	35	6.4
18–20	179	32.7
21–23	254	46.4
≥24	79	14.4
Hometown		
Guiyang	495	90.5
Other cities	52	9.5
Ethnicity		
Han	386	70.6
Others	161	29.4
Stage of study		
Junior college/undergraduate	501	91.6
Master/Doctor	46	8.4
Major		
Medical	64	11.7
Non-medical	483	88.3
Living arrangement		
On-campus	452	82.6
Off-campus	95	17.4
Living expenses per month (¥)		
<2,000	462	84.5
≥2,000	85	15.5
Student loans		
No	472	86.3
Yes	75	13.7

## Results

### Description of socio-demographic information

A total number of 547 valid questionnaires were collected in this study. Table 1 presents the distribution of socio-demographic information of the participants. Among the four age groups, the highest proportion of participants (254, 46.4%) was in the 21–23 age group. The majority (495, 90.5%) of the participants were from Guiyang City, and 386 (70.6%) participants were Han ethnic. There were 501 participants (91.6%) studying in junior college or undergraduate programs. Among all participants, 483 (88.3%) majored in non-medical professions, 452 (82.6%) lived on-campus, 462 (84.5%) had monthly living expenses of <¥2,000, and 472 (86.3%) did not have student loans.

### Descriptive statistics and correlation analysis

Table 2 shows the mean scores and SDs for depression, anxiety, social support, and self-identity were 10.06 ± 10.09, 46.35 ± 9.21, 33.09 ± 7.27, and 27.48 ± 5.71, respectively. Among all participants, 38.39% (210 of 547) were depressed and 28.34% (155 of 547) were anxious. Meanwhile, Table 2 displays Spearman correlations among depression, anxiety, social support, and self-identity. The results indicated that depression was positively correlated with anxiety (*r* = 0.180), while negatively correlated with social support (*r* = −0.217) and self-identity (*r* = −0.316). Anxiety was negatively associated with social support (*r* = −0.249) and self-identity (*r* = −0.282). In addition, social support had a positive correlation with self-identity (*r* = 0.241). All *P* values were <0.001.

**Table 2:** Descriptive statistics and correlation analysis results.

Variable	Mean	SD	Depression	Anxiety	Social support	Self-identity
Depression	10.06	10.09	1			
Anxiety	46.35	9.21	0.180^*^	1		
Social support	33.09	7.27	−0.217^*^	−0.249^*^	1	
Self-identity	27.48	5.71	−0.316^*^	−0.282^*^	0.241^*^	1

SD, standard deviation.

^*^P <0.001.

### Test of study models

The model fit indices of the SEM in Figure 2 were satisfactory (χ^2^/df = 1.287, RMSEA = 0.023, AGFI = 0.886, IFI = 0.977, NFI = 0.906, TLI = 0.974, CFI = 0.977, GFI = 0.904).

**Figure 2:**
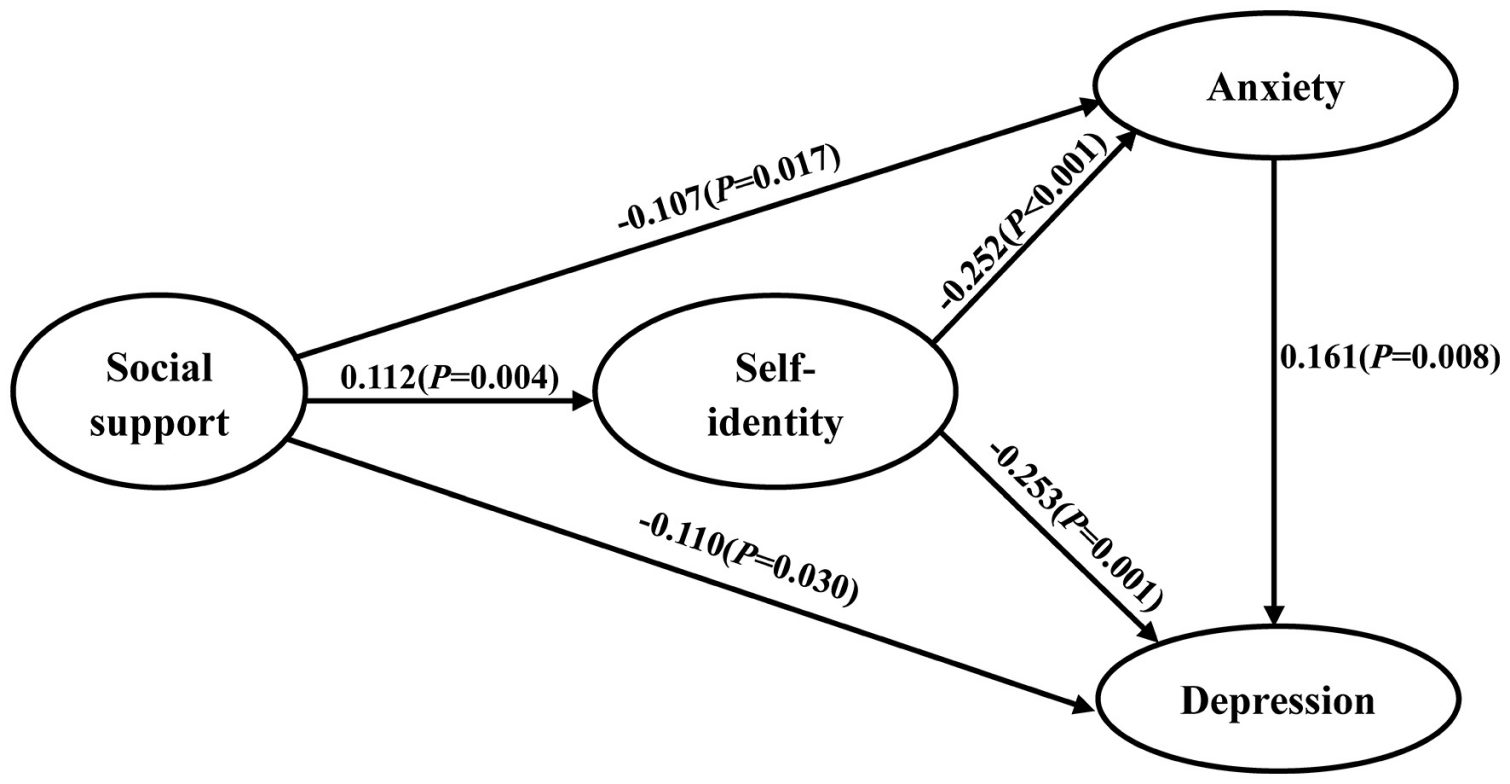
Results of structural equation model of associations among social support, self-identity, depression, and anxiety in college student MSM.

The results of SEM are tabulated in Table 3. We found that depression was associated with reduced social support (β = −0.110, *P* = 0.030) and self-identity (β = −0.253, *P* = 0.001), but associated with increased anxiety (β = 0.161, *P* = 0.008). Lower levels of social support and self-identity were significantly associated with higher anxiety, with β = −0.107 (*P* = 0.017) for social support and β = −0.252 (*P* < 0.001) for self-identity. Also, social support was positively associated with self-identity (β = 0.112, *P* = 0.004).

**Table 3:** Results of structural equation modeling.

Outcome variables	Predict variables	β	*SE*	*t*	*P*
Depression	Social support	−0.110	0.051	−2.166	0.030
Depression	Self-identity	−0.253	0.079	−3.227	0.001
Depression	Anxiety	0.161	0.061	2.663	0.008
Anxiety	Social support	−0.107	0.045	−2.388	0.017
Anxiety	Self-identity	−0.252	0.070	−3.580	<0.001
Self-identity	Social support	0.112	0.039	2.685	0.004

SE, standard error.

### The mediating role of self-identity

Table 4 presents the results of the mediation analyses examining whether self-identity mediated the associations of social support with both depression and anxiety. The results showed that self-identity served as a mediator in the relationship between social support and depression (β = −0.028, *P* = 0.010). Additionally, self-identity also played a mediating role in the relationship between social support and anxiety (β = −0.028, *P* = 0.011).

**Table 4:** Mediating role of self-identity in the associations of social support with depression and anxiety among all participants.

Mediation pathways	Coefficient^*^(95% *CI*)	*P*
Social support → Self-identity → Depression	−0.028 (−0.140, −0.007)	0.010
Social support → Self-identity → Anxiety	−0.028 (−0.135, −0.007)	0.011

CI, confidence interval.

^*^Unstandardized coefficient.

## Discussion

Our findings indicated that college student MSM in Guiyang, China exhibited mild depression (Moon et al., 2017) and mild-to-moderate anxiety (Zung, 1986). Social support and self-identity were negatively associated with anxiety and depression among college student MSM. Furthermore, self-identity significantly mediated the associations of social support with both depression and anxiety.

### Depression and anxiety among college student MSM

Compared to the reported prevalence rates of depression (43.2%) and anxiety (32.2%) among the general Chinese MSM population, the present study found lower prevalence rates of depression (38.39%) and anxiety (28.34%) among college student MSM in Guiyang (Wei et al., 2020). This difference may be explained by several factors. First, Resilience theory suggests that younger individuals, such as college students, generally exhibit higher psychological resilience, which may serve as a protective buffer against depression and anxiety. Psychological resilience tends to decline with age and prolonged exposure to social stress, which may contribute to increased depression and anxiety in older populations (Masten, 2018). Second, higher education could promote tolerance of sexual minorities (Ohlander et al., 2005). Previous studies have indicated that higher education is usually accompanied by more diverse social science education (such as gender studies and psychology), which may promote understanding and acceptance of sexual minorities and reduce prejudice, thereby reducing the prevalence of depression and anxiety (Herek, 2002; Ohlander et al., 2005).

### Associations of social support with both depression and anxiety

Consistent with previous studies conducted among sexual minority college students in other regions of China (Li et al., 2023; Xie et al., 2024), the results of our study showed that social support was negatively linked to anxiety and depression among college student MSM. Several theories could explain this finding. The Minority Stress Theory suggests that MSM usually face various stressors such as social prejudice and stigma, which are associated with several mental health problems (Meyer, 2003). The Stress Buffering Hypothesis indicates that social support could effectively buffer against pressure caused by these stressors by reducing perceived stress and loneliness, improving self-acceptance, psychological resilience, and coping resources, thereby reducing the risk of depression and anxiety (Cohen and Wills, 1985; Mcdavitt et al., 2008; Shilo and Savaya, 2011).

### Associations of self-identity with both depression and anxiety

This study found that self-identity was negatively associated with depression and anxiety among college student MSM, which was consistent with previous findings in other populations (Chen et al., 2021; Li et al., 2022). There are two possible reasons for this phenomenon. Firstly, a high level of self-identity may reduce the internal stigma, cognitive burden, and behavioral burden among college student MSM (Chan et al., 2020; Mi et al., 2022; Ummak et al., 2023). An intervention study showed that MSM who received self-confidence training and self-affirmation techniques were able to restructure their cognitions of minority stress, thereby reducing their risk of depression and anxiety (Pachankis, 2014). Secondly, a high level of self-identity may help individuals to adopt positive coping strategies to reduce the risk of depression and anxiety when facing stressors, such as discrimination and prejudice (Zhou et al., 2021). For example, when young MSM are exposed to prejudice, they choose to ignore it or vent their negative emotions through crying and talking (Mcdavitt et al., 2008).

### Association between anxiety and depression

Our study found a positive link between anxiety and depression among college student MSM, which was consistent with existing literature. Previous studies also found that anxiety and depression were positively correlated in various populations, including sexual minority college students (Luo et al., 2025), elderly patients with hypertension (Ji et al., 2021), and residents living in mega-communities (Lu et al., 2024). The Response Style Theory could explain this finding. This theory suggests that anxious individuals are more likely to induce rumination, which ultimately aggravates depressive symptoms by frequently recalling past negative events, inhibiting problem-solving abilities, and interfering with instrumental behavior (Nolen-Hoeksema et al., 2008). A previous study indicated that rumination due to prejudice events or internalized stigma might increase the level of depression among sexual minorities (Timmins et al., 2020). Another potential explanation was that long-term anxiety due to multiple stressors (such as financial pressures and discrimination) could lead to insomnia, fatigue, or individuals' emotion regulation disorder, ultimately developing into depression (Mesurado et al., 2018; Parchem et al., 2024).

### Mediating role of self-identity

The present study revealed that self-identity played a mediating role between social support and depression status among college student MSM, which was in line with a previous study carried out among general college students in China (Li et al., 2022). One possible explanation is that the internalization of social support positively shapes an individual's self-perception, ultimately leading to fewer depressive symptoms (Pan et al., 2022). Previous studies have suggested that social support from peers, families, or the LGBT communities might promote a positive perception of identity, enabling MSM individuals to accept their sexual orientation, reduce internalized shame, and enhance confidence to prevent depression (Meyer, 2003; Kertzner et al., 2009; Ryan et al., 2010).

Our results also showed that self-identity mediated the relationship between social support and anxiety among college student MSM, which is consistent with the widely recognized protective role of identity integration in mental health (Guo et al., 2025). According to Minority Stress Theory, sexual minorities often face discrimination and stigma, which can increase their vigilance and uncertainty about future threats, both of which are closely linked to anxiety (Meyer, 2003). Social support from friends, family, or the LGBT community can buffer these negative effects by providing emotional comfort and practical help, enabling individuals to better cope with stress and view their experiences in a less threatening way (Cohen and Wills, 1985). More importantly, when social support is internalized, it helps individuals develop a positive self-identity. Identity development theory suggests that forming a clear and valued sexual identity is a critical step toward psychological wellbeing (Cass, 1979). A strong and positive self-identity can reduce internalized shame and improve self-esteem, which in turn lowers the excessive worry and vigilance commonly seen in anxiety (Sun et al., 2020). Taken together, these findings suggest that social support reduces anxiety both directly and indirectly by strengthening self-identity. These highlights the need for interventions that not only build supportive networks but also actively promote identity acceptance among college student MSM.

### Limitations

There are several limitations in this study. Firstly, the cross-sectional design of our study has an inherent limitation in inferring causal relationships among social support, self-identity, depression, and anxiety. Secondly, since college student MSM are hidden, it is difficult to perform random sampling among this population. Thus, the sample of our study might not be well representative of the whole college student MSM, and the generalizability of our findings might be limited. Lastly, all data in this study were derived from self-reported questionnaires. Therefore, social desirability bias and recall bias could not be fully controlled, especially regarding sexual behavior characteristics and mental health symptoms. Although participants were assured of anonymity to encourage honest responses, they may still have underreported or overreported sensitive information, or may have had difficulty accurately recalling past experiences. These inherent limitations of self-report measures should be considered when interpreting the results of this study.

## Conclusions

Our study examines the associations among social support, self-identity, depression, and anxiety in college student MSM. In conclusion, college student MSM in Guiyang City were mildly depressed and mildly to moderately anxious, and compared with general Chinese MSM, the levels of anxiety and depression in college student MSM were lower. Social support and self-identity were negatively associated with anxiety and depression. Self-identity significantly mediated the associations of social support with both depression and anxiety. These findings suggest that interventions targeting depression and anxiety among college student MSM are still necessary, and the interventions can be combined with content relevant to enhance social support and self-identity. Future studies are needed to focus on longitudinal data to investigate the causality among social support, self-identity, depression, and anxiety in college student MSM.

## Data Availability

The raw data supporting the conclusions of this article will be made available by the authors, without undue reservation.
