# Supplementary material for: Association among social support, self-identity, depression, and anxiety in college student men who have sex with men in Guiyang, China
Source: Front Psychol. 2026 Oct 8;17:1911298. doi: 10.3389/fpsyg.2026.1911298 (PMC13687410; doi:10.3389/fpsyg.2026.1911298)
Supplement: Supplementary file 1 [file Table_1.docx]

**Supplementary Table 1 The Self-identity as Homosexual Scale**

| **Item** | **Statement** |
| --- | --- |
| 1 | Sometimes I wish I were not gay |
| 2 | Sometimes I think that if I were straight, I would be happier |
| 3 | In order to avoid disclosure of my homosexuality, I have tried not to be attracted by men |
| 4 | Sometimes I wish I could become more sexually attractive to women |
| 5 | I feel that being gay is a personal shortcoming for men |
| 6 | Sometimes, I feel ashamed of my sexual orientation |
| 7 | I am afraid my family and friends will find out my sexual orientation |
| 8 | I try to look and act masculine in order to avoid others rejection |
| 9 | I am happy that I am gay |

1 = Strongly Disagree, 2 = Disagree, 3 = Neutral, 4 = Agree, 5 = Strongly Agree (Item 9 is reverse scored).
